# NIDS-Mamba: Lightweight Network Intrusion Detection for IoT Sensor Networks via State Space Models

**DOI:** 10.3390/s26092766

**Published:** 2026-04-29

**Authors:** Zixiang Ding, Jiahao Zheng, Xianyun Wu

**Affiliations:** 1 School of Optoelectronic Engineering, Xidian University, Xi’an 710071, China; 23009300645@stu.xidian.edu.cn; 2 School of Physics, Xidian University, Xi’an 710071, China; 23009300632@stu.xidian.edu.cn; 3 State Key Laboratory of Integrated Services Networks, Xidian University, Xi’an 710071, China

**Keywords:** IoT, security, edge computing, network intrusion detection, Mamba

## Abstract

The ubiquity of resource-constrained Internet-of Things (IoT) nodes creates an urgent demand for network intrusion detection systems (NIDSs) optimized for edge devices with limited computing power. In this paper, we propose a new NIDS system based on Mamba. NIDS-Mamba uses a dynamic sparse attention and a lightweight state space to jointly learn from short-term anomaly and long-term attack patterns. We use standardized NF-UNSW-NB15 and NF-CSE-CIC-IDS2018 datasets to verify the effectiveness of this NIDS-Mamba model. We find that this NIDS-Mamba model is very effective in dealing with extreme class imbalance problems. In the NF-CSE-CIC-IDS2018 dataset, the model achieves 98.32% accuracy, 96.98% F1-score, and an AUC of 0.9996. Most notably, the model is very robust in handling extreme class imbalance problems in the NF-UNSW-NB15 dataset. It achieves 97.03% G-Mean, 0.7915 MCC, and 0.9983 AUC, far exceeding other baseline models. Compared to Transformer-based baselines, NIDS-Mamba achieves nearly an order-of-magnitude improvement in throughput while maintaining a parameter footprint compatible with edge deployment constraints. The proposed architecture effectively mitigates the quadratic complexity and memory wall inherent in standard Transformers, ensuring compatibility with Limited RAM and strict energy constraints. The proposed model achieves a compact design with 1.12 million parameters and a peak inference memory of 5.4 MB, ensuring its feasibility for edge-based IoT nodes. These properties make NIDS-Mamba a strong candidate for deployment on IoT gateways and edge sensor nodes in smart home, industrial IoT, and critical infrastructure scenarios.

## 1. Introduction

The rapid proliferation of the Internet of Things (IoT) has led to an unprecedented expansion of interconnected sensor networks, with billions of devices deployed across smart cities, industrial control systems, and critical infrastructure. El País reports that connected devices will exceed 25 billion in early 2026, heading toward 40 billion before 2030 [1]. However, this massive deployment has also significantly enlarged the attack surface of networked systems [2]. IoT and sensor networks are inherently vulnerable due to their heterogeneous architectures, always-on connectivity, and minimal onboard security mechanisms [3]. Many devices operate with limited computational capability and lack robust protection against cyber threats, making them prime targets for large-scale attacks such as botnets, distributed denial-of-service (DDoS), and data exfiltration [4,5,6]. Indeed, recent cybersecurity analyses indicate a sharp rise in IoT-targeted attacks, with hundreds of millions of intrusion attempts reported annually on edge-connected infrastructure [7]. These challenges place IoT security—particularly within resource-constrained sensor environments—at the forefront of modern network intrusion detection research.

Currently, deep learning-based network intrusion detection systems (NIDSs) have made remarkable advancements in three main dimensions: (1) the capability to achieve superior detection accuracy on large-scale benchmark datasets; (2) the ability to detect complex and unseen attacks such as zero-day and polymorphic attacks; (3) reduction in the effort required for manual feature engineering via end-to-end representation learning [8,9,10,11]. These advances highlight the fundamental advantages of deep learning-based NIDS over traditional approaches, such as Random Forest and Support Vector Machines [8,9]. Unlike conventional approaches that require extensive manual feature engineering and are often constrained by predefined rules, deep learning models—such as Convolutional Neural Networks (CNNs) and Long Short-Term Memorys (LSTMs)—utilize hierarchical architectures to automatically learn intricate, high-dimensional feature representations directly from raw network traffic or flow-based data [10,11]. This capability for automated feature extraction significantly enhances the detection of sophisticated, polymorphic, and zero-day attacks that traditional methods frequently fail to identify [12].

Despite significant advances in deep learning-based NIDS, existing approaches struggle to meet the stringent requirements of IoT and edge environments. This limitation stems from two fundamental challenges. First, Transformer-based models, while powerful in capturing long-range dependencies, suffer from quadratic computational complexity with respect to sequence length, resulting in excessive memory consumption and energy usage [13]. Such characteristics make them impractical for deployment on IoT-based microcontrollers, resource-constrained embedded systems, or low-power IoT gateways. Second, recurrent architectures such as recurrent neural networks (RNNs) and LSTM networks exhibit inherent sequential processing bottlenecks, which severely limit throughput and hinder real-time detection in high-speed network scenarios [14,15]. More critically, existing studies often overlook the strict energy budgets and limited RAM availability of edge devices [16,17], where even moderate model sizes can exceed hardware constraints [18,19]. Resource limitations mainly manifest in three aspects: (1) Storage Memory, which limits the total parameter footprint to fit within the few-megabyte Flash capacity of typical embedded systems; (2) Runtime RAM, which demands minimal peak memory usage during inference to prevent Out-of-Memory (OOM) errors, particularly those caused by the quadratic activation growth seen in traditional attention mechanisms; and (3) energy budget, which necessitates low computational complexity (FLOPs) to extend the operational lifespan of battery-driven IoT sensor nodes.

In conclusion, while the current deep learning-based NIDSs have shown powerful detection performance, they are not tailored to IoT environments with deployment constraints. A substantial gap exists in model effectiveness and deployment desirability in terms of detection accuracy, computing cost, and resource consumption.

As a result, there exists a clear gap between high-performance NIDS models and their feasibility in real-world IoT sensor deployments. To address the aforementioned hardware constraints and the inherent trade-off between modeling efficiency and detection fine-grainedness, the optimization goal of this paper is to achieve state-of-the-art detection accuracy while simultaneously maximizing inference throughput and minimizing the hardware resource footprint, including peak RAM, storage flash, and computational complexity (FLOPs).

In real-world IoT applications, not only high detection accuracy but also system-level constraints enforced by edge environments are prerequisites for NIDS. Recent studies have revealed that the memory capacity, computational ability, and energy budget of IoT devices are severely constrained, which essentially hinders the application of complex DL-based methods [16,17,18,19]. Therefore, the NIDS proposed in this paper needs to be capable of adapting to the heterogeneous hardware layers that are commonly found in the Internet of Things environment, ranging from edge gateways (e.g., NVIDIA Jetson) to high-performance single-board computers (e.g., Raspberry Pi 4B). Within these environments, the specific resource limitations addressed in this work include the restricted RAM availability (often within a few megabytes), the limited on-chip flash memory for model storage, and the stringent energy budgets that preclude the use of power-intensive hardware accelerators. By defining these boundaries, NIDS-Mamba is designed to provide linear-time detection efficiency without exceeding the physical constraints of such decentralized sensor nodes. Therefore, these constraints naturally impose additional design requirements on NIDS, including low memory footprint, high throughput, and energy efficiency, which are essential for real-world deployment rather than optional design choices.

In real-world IoT environments, network traffic is characterized by both complex topological structures and high-velocity data flows. The complex topology introduces intricate dependencies between distributed devices, while high-speed traffic generates long and dense temporal sequences. Together, these characteristics significantly increase the difficulty of accurately modeling network behavior in real time. This creates a critical challenge: NIDSs must simultaneously capture relational dependencies across devices and temporal patterns within high-speed flows, under strict resource constraints. For instance, graph neural networks (GNNs) have demonstrated exceptional capability in capturing the relational dependencies between communication entities by modeling network traffic as graph structures [20]. Simultaneously, Temporal Convolutional Networks (TCNs) have emerged as a robust alternative for sequence modeling, offering better parallelism than LSTMs while maintaining a stable gradient flow through dilated convolutions [21,22]. Despite these advances, achieving a balance between the high-dimensional relational capture of GNNs and the linear-time efficiency required for IoT edge deployment remains a significant challenge.

Recent developments in state space models (SSMs), particularly the Mamba architecture, offer a promising alternative for efficient sequence modeling. The relevance of sequence modeling to NIDSs lies in the temporal nature of network communication; an intrusion is rarely a single isolated event but rather a series of interrelated packets whose malicious intent is only identifiable through their temporal correlations. In the IoT ecosystem, the challenge of security is intrinsically a problem of finding efficient sequence modeling. Since IoT gateways must process high-velocity traffic flows with extremely limited Static-Random-Access-Memory (SRAM) and power budgets, traditional models with quadratic complexity (like Transformers) create a “memory wall”, while sequential models (like LSTMs) suffer from high latency. Therefore, the core of IoT security research has shifted toward identifying sequence modeling paradigms that can capture long-range attack patterns with linear-time efficiency. Unlike traditional RNNs and Transformers, Mamba achieves near-linear computational complexity while maintaining strong capability in modeling long-range dependencies [23,24]. This property enables the model to process network traffic efficiently while focusing on critical temporal patterns, such as burst behaviors in Denial-of-Service attacks. The direct correlation between network traffic and large-scale IoT deployments stems from the fact that IoT sensor nodes typically generate high-frequency, telemetry-driven traffic flows, where any deviation in communication frequency or payload size serves as a primary indicator of device compromise [13]. In massive IoT ecosystems, the sheer volume of concurrent flows necessitates a model capable of linear-time complexity to prevent a “processing wall,” ensuring that the identification of temporal anomalies can be performed locally at the edge without introducing prohibitive latency into the network fabric [24,25]. Importantly, the linear complexity of Mamba not only improves throughput but also reduces computational overhead and energy consumption, making it particularly suitable for deployment on resource-constrained edge devices [25].

The application of Mamba in network intrusion detection remains in its early stages [26]. While Mamba effectively mitigates the quadratic complexity of Transformers, it faces an “information bottleneck” where a fixed-size state may struggle to preserve fine-grained historical details of stealthy attacks. Recognizing that Mamba and Attention mechanisms possess complementary expressive power, this paper proposes a novel hybrid deep learning architecture, termed NIDS-Mamba. The model integrates a lightweight state space module with dynamic sparse attention and depthwise separable convolution. This synergy allows the Mamba blocks to handle long-range filtering with linear efficiency, while the dynamic sparse attention mechanism restores critical global dependencies without the prohibitive cost of standard Transformers. By leveraging the lightweight nature of flow-based statistical features, the architecture achieves a balance between detection accuracy and computational efficiency, making it well suited for real-time network environments.

The contributions of this paper can be multifaceted. For one, we have successfully created a highly efficient NIDS that operates at the flow level, where network traffic is aggregated into bidirectional flows identified by the standard 5-tuple to extract statistical features for threat detection. In the process, we preserve user privacy by design by utilizing flow-level metadata rather than raw packet payloads. Second, we have proposed a new synergistic mechanism that incorporates the selective state space model, attention mechanism, and convolutional mechanism to overcome the intrinsic recall limitations of pure SSMs. The designed mechanism filters irrelevant information while retaining critical spatial–temporal features of short-term anomalies and long-term attack patterns by exploiting the global receptive field of attention layers. Third, we demonstrate that NIDS-Mamba provides a comprehensive solution to the “accuracy–resource” trade-off. Specifically, it achieves competitive high-performance detection while maintaining a minimal RAM footprint and a low energy budget, making it intrinsically compatible with the strict physical constraints of edge sensor nodes. To address these challenges, this paper proposes a lightweight and efficient NIDS architecture, termed NIDS-Mamba, which is specifically designed for resource-constrained IoT environments.

## 2. Flow-Based Anomaly Detection

### 2.1. Flow-Based Anomaly Detection Framework

Before detailing the proposed architecture, it is essential to define the “flow-based” detection paradigm and the specific problem it addresses. Unlike packet-level NIDSs that perform deep inspection of individual packet payloads, flow-based detection analyzes network traffic as a series of bidirectional communication records. A network “flow” is defined by its 5-tuple (source/destination IP, source/destination port, and protocol) and is characterized by aggregated statistical features, including duration, packet counts, and byte rates.

From a problem formulation perspective, we treat network intrusion detection as a sequence classification task. Formally, given a sequence of *L* standardized flow feature vectors $X=\{x_{1},x_{2},\ldots ,x_{L}\}$, where each $x_{i}\in R^{D}$ represents the statistical attributes of a flow at time step *i*, the objective is to learn a mapping function f: X →Y. Here, Y denotes the predicted label, which can be binary (benign or malicious) or multiclass (specific attack categories). This framework is designed to solve the sequence modeling problem by first condensing high-dimensional raw traffic into manageable flow vectors, and then employing the NIDS-Mamba module to capture both local anomalies and long-term attack patterns with linear-time efficiency—a critical requirement for resource-constrained edge gateways.

**Figure 1:**
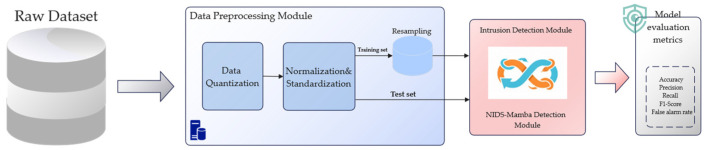
The architecture of the proposed flow-based anomaly detection framework.

As illustrated in Figure 1, the flow-based anomaly detection framework primarily consists of three components: the data preprocessing module, the intrusion detection module, and the performance evaluation module. The data preprocessing module transforms raw traffic data into an input format compatible with neural networks through operations such as discretization, standardization, normalization, and resampling. The intrusion detection module incorporates the proposed NIDS-Mamba model; by analyzing traffic features from multiple perspectives, it effectively identifies anomalous behaviors within complex data characterized by long-term temporal dependencies. Finally, the detection performance is rigorously assessed via the evaluation module using comprehensive metrics including accuracy, precision, recall, false positive rate (FPR), F1-score, G-Mean, Matthews Correlation Coefficient (MCC), and Area Under the Curve (AUC). As a threshold-independent metric, AUC is specifically incorporated to provide a holistic evaluation of the model’s discriminative capability. This is particularly vital for the imbalanced network traffic datasets used in this study, as it measures the model’s ability to distinguish between benign and malicious flows regardless of the specific classification threshold.

### 2.2. Data Preprocessing

Data preprocessing aims to optimize model performance by ensuring data balance and consistency through quantization, standardization, and normalization.

#### 2.2.1. Data Quantization

Besides numerical values such as Total Packets and Duration, flow data consists of categorical values such as Type of Service and Connection State. These values are converted to numerical values using LabelEncoder() from Scikit-learn. Moreover, benign and attack data are differentiated by their category values, benign and attack. Benign and attack data are represented by 0 and 1 in binary classification, whereas in multi-classification, One-Hot Encoding is used to convert different types of attack data into unique binary vectors.

#### 2.2.2. Standardization & Normalization

Standardization is applied to scale features to a uniform variance, preventing features with larger magnitudes from dominating the loss function. The standardization formula is represented by Equation (1):

(1) $$z=\frac{x-\mu }{\sigma }$$

In the above equation, *x* represents the raw data point, *μ* denotes the mean, *σ* is the standard deviation, and *z* refers to the standardized data.

Normalization maps the range of values to a fixed range of [0, 1]. This technique helps the model process all features in the dataset at the same scale. In this technique, a linear transformation of the dataset is performed using the maximum and minimum values. This technique limits the range of the dataset to a specific range. Normalization can be represented by the formula given in Equation (2):

(2) $$x_{norm}=\frac{x-\min\left(X\right)}{\max\left(X\right)-\min\left(X\right)}$$

where *x* is the raw data, min(*X*) and max(*X*) represent the minimum and maximum values of the feature, respectively, and *x_norm_* is the normalized data.

#### 2.2.3. Resampling

In network traffic datasets, class imbalance problems may occur, where the number of benign network traffic samples greatly exceeds the number of attack network traffic samples. Oversampling the minority class will balance the dataset to some extent. In this section, the Synthetic-Minority Oversampling Technique (SMOTE) algorithm will be used to oversample the minority class [27,28,29]. In the SMOTE algorithm, new samples are created based on the neighborhood of minority class samples. These new samples created in the algorithm can be referred to as “synthetic” samples. Therefore, overfitting can be avoided to some extent. The process of the SMOTE algorithm can be described as follows:

Randomly select a sample *x_i_* from the minority class samples (e.g., attack samples), and select one or more nearest neighbors for the sample *x_i_*. Let the nearest neighbors of *x_i_* be (*x_i_*_,1_*, x_i_*_,2_, …, *x_i_*_,*m*_), where *m* is the number of neighbors selected. For each neighbor *x_i_*_,*j*_*,* generate a new synthetic sample *x_new_* by interpolating between *x_i_* and *x_i_*_,*j*_. The formula is expressed in Equation (3):

(3) $$x_{new}=x_{i}+\lambda \cdot \left(x_{i,j}-x_{i}\right)$$

where *λ* is a coefficient randomly selected within the interval [0, 1] to control the degree of interpolation.

### 2.3. Detection Model Based on NIDS-Mamba

According to the TCP/IP network protocol, network packets are composed of multiple traffic bytes, which are further combined to constitute data flows. In this way, the bytes, packets, sessions, and their combinations in the network traffic can be compared to the combination of characters, words, sentences, and texts in natural language. Therefore, traffic data can essentially be regarded as sequential data with strong contextual dependencies. From this perspective, network intrusion detection can be reformulated as a sequence labeling or classification task, where the model must read the flow to identify semantic anomalies. The requirement for efficient sequence modeling arises directly from the hardware limitations of decentralized sensor nodes. In these environments, the window of observation for a flow often needs to be long to detect stealthy multi-stage attacks, yet the peak memory must remain at the low-megabyte level. Efficient sequence modeling, specifically with linear complexity $O\left(L\right)$, is the only viable path to enable these resource-constrained nodes to perform real-time deep packet or flow-based analysis without offloading to a centralized cloud. Theoretically, since power consumption in embedded AI is primarily driven by computational density and memory data movement, the reduction from quadratic $O\left(L^{2}\right)$ to linear complexity $O\left(L\cdot s_{n}\right)$ inherently minimizes the energy budget required per inference. This linear scaling ensures that the energy-per-flow remains stable even as the observation window for detecting stealthy attacks expands. However, current machine learning-based NIDSs mainly focus on classifying individual network flow records, without considering the temporal dependencies and long-term interaction characteristics present in network traffic during communication.

Despite their efficacy in long-term dependency capture, attention-based NIDSs are often impractical for edge session analysis due to the $O\left(L^{2}\right)$ computational overhead previously identified. Furthermore, the Transformer’s global attention mechanism may lead to scattered attention distributions when dealing with highly redundant or noisy network traffic, thereby reducing the model’s ability to focus on critical attack features.

Meanwhile, while the Mamba model based on Selective SSM is theoretically capable of modeling long-sequence dependencies with linear complexity and demonstrates superior efficiency and scalability in long-context tasks, its implicit attention mechanism is difficult to interpret directly. This limits the model’s interpretability, debuggability, and behavioral analysis in security scenarios. Furthermore, when faced with complex multi-stage attacks or cross-session correlated features, the implicit information flow within Mamba’s selective mechanism may be difficult to directly control or constrain, thereby affecting the model’s reliability in security-critical tasks [3].

**Figure 2:**
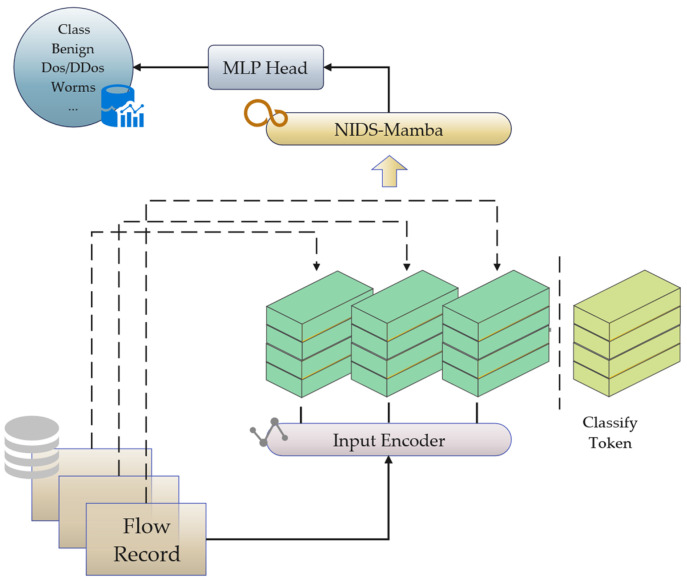
Structure of the NIDS-Mamba model.

To address these issues, this paper proposes the NIDS-Mamba neural network model, which integrates dynamic sparse attention with lightweight state space modules to exploit their complementary expressive power [30]. This hybrid architecture leverages the selective SSM to efficiently model long-range temporal dependencies in linear time, while utilizing the attention mechanism to attend to the entire sequence simultaneously, thereby compensating for the potential information loss caused by Mamba’s state compression. This synergy allows the model to jointly capture short-term anomaly features and long-term attack behavior patterns, improving the overall performance of network traffic detection. The architecture of the proposed model is illustrated in Figure 2 and consists of three main components: the input encoder, the NIDS-Mamba Block, and the Multilayer Perceptron (MLP) Head. The input encoder transforms network traffic flows into fixed-length vectors. After the input sequence is vectorized, a learnable Classify Token is appended to support the subsequent classification task. Finally, an MLP head processes the output sequence generated by the NIDS-Mamba block to convert context embeddings into final predictions for intrusion classes.

#### 2.3.1. Input Encoder

The input encoder in flow-based network intrusion detection systems is considered to be an essential component in the conversion of raw flow data into feature vectors, which can be further processed by the NIDS-Mamba model. Flow data is usually presented in tabular form, with each flow consisting of fixed fields that can be categorized into two types: numerical fields, such as the number of packets, in which the numerical differences have contextual significance, and categorical fields, such as ports, in which the numerical differences do not have any significance. For instance, ports 25 and 22 have significant differences, as they belong to different protocols, while ports 443 and 8080 have similar uses.

**Figure 3:**
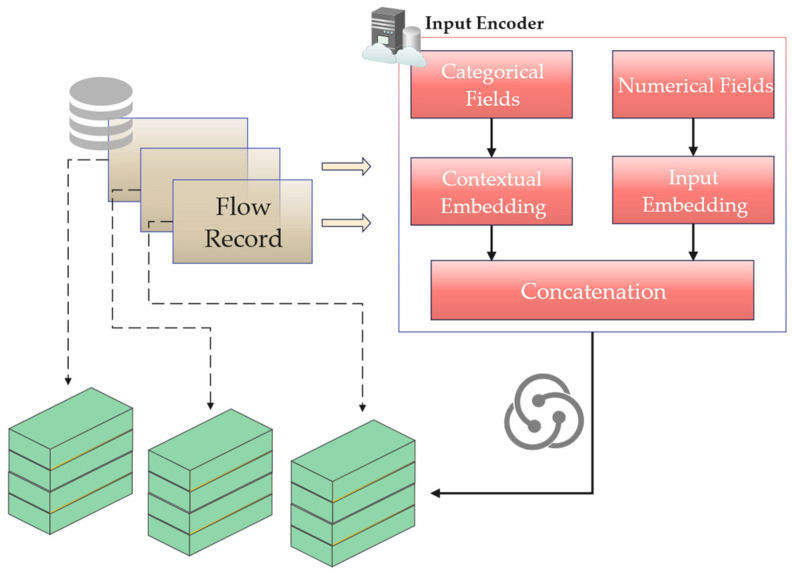
Diagram of the input encoding module for numerical and categorical features (reproduced with permission from [31]).

Therefore, the input encoder must process categorical fields such that their representation in the vector space is not influenced by numerical magnitude, ensuring that the generated feature vectors reflect key information related to network traffic for subsequent model analysis. This section encodes categorical fields by converting them into continuous vectors with contextual meaning separately [10,31], then concatenating these vectors with numerical vectors to obtain a feature vector available for the subsequent model. Since there is no inherent order among features in tabular data, position encoding is not employed. The process is illustrated in Figure 3.

Assume that (*x_cat_*, *x_num_*) represents a feature target pair, where *x_cat_* denotes all categorical features and *x_num_* represents all numerical features. *x_cat_* = {*x*_1_, *x*_2_, …, *x_m_*} where each *x_i_* represents a categorical field, and $i\in \left\{1,\cdots ,m\right\}$. The value of *x_i_* belongs to a finite set. Through an embedding layer, a *d*-dimensional continuous vector $e_{x_{i}}$ is learned for each category value, as shown in Equation (4):

(4) $$e_{x_{i}}=\mathrm{Embedding}\left(x_{i}\right)$$

The embedding layer here is designed as a lookup table, where each *x_i_* corresponds to a vector $e_{x_{i}}$. The parameters of the embedding layer consist of an embedding matrix $E\in R^{K\times d}$, where *K* denotes the total number of possible category values. The representation is shown in Equation (5):

(5) $$E=\left[\begin{array}{c} e_{x_{1}}\\ e_{x_{2}}\\ \vdots \\ e_{x_{K}} \end{array}\right]$$

The parameters of the embedding layer are continuously updated during the training process to ensure that similar categories have closer representations in the embedding space. Given a specific value *k* for *x_i_*, its corresponding embedding vector can be obtained by retrieving the *k*-th row of the embedding matrix, as expressed in Equation (6):

(6) $$e_{x_{i}}=E_{k}$$

This denotes the retrieval of the corresponding embedding vector $e_{x_{i}}$ for the categorical value $x_{i}$ from the embedding matrix.

After concatenation of categorical and numerical embeddings, the input flow record is mapped to a fixed hidden dimension of *D* = 128. This dimension ensures a sufficiently high-order representation of network features while remaining lightweight for edge gateways.

#### 2.3.2. NIDS-Mamba Model Architecture

**Figure 4:**
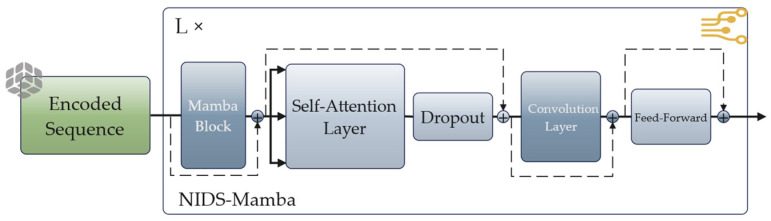
Structure of the NIDS-Mamba module. The plus sign represents the residual connection.

As illustrated in Figure 4, the overall architecture of NIDS-Mamba is a hierarchical stacking of four identical layers, where each layer integrates a Mamba block, a self-attention layer, a Feed-Forward Network (FFN), and a convolutional layer through a synergistic interaction mechanism. Within each layer, these components operate in a sequential and complementary manner to extract multi-level features from network flows.

First, the Mamba block carries out an initial selective scan on the input feature vector to obtain time-varying dependence and exclude irrelevant noises to establish a more refined representation as the basis for further processing. After this step, the result will be fed into the self-attention layer, where the model will apply the attention mechanism to learn long-term temporal dependencies and high-level correlation within the multidimensional feature vectors. The self-attention layer makes up for the possible information loss during the dimension reduction of the SSM layer by enhancing the modeling ability and learning the correlation between different features. Then, the next step would be the feeding of the feature vector to the FFN for non-linear transformation of the aggregated data for mapping to a new feature space for learning complicated relationships necessary for traffic classification. In the end, convolutional layers enable a larger field for dependency learning for both local and global interactions among tokens in the traffic flows. Throughout this four-layer process, residual connections are employed between each module to address gradient vanishing and exploding problems, ensuring that critical information from the original embedding flows smoothly through the deep architecture.

To ensure the reproducibility of the proposed NIDS-Mamba, the detailed architectural configurations and hyperparameter settings are summarized in Table 1. These parameters were determined through empirical optimization and grid search to balance detection accuracy and computational overhead for IoT edge deployment.

a. Mamba-Block

**Figure 5:**
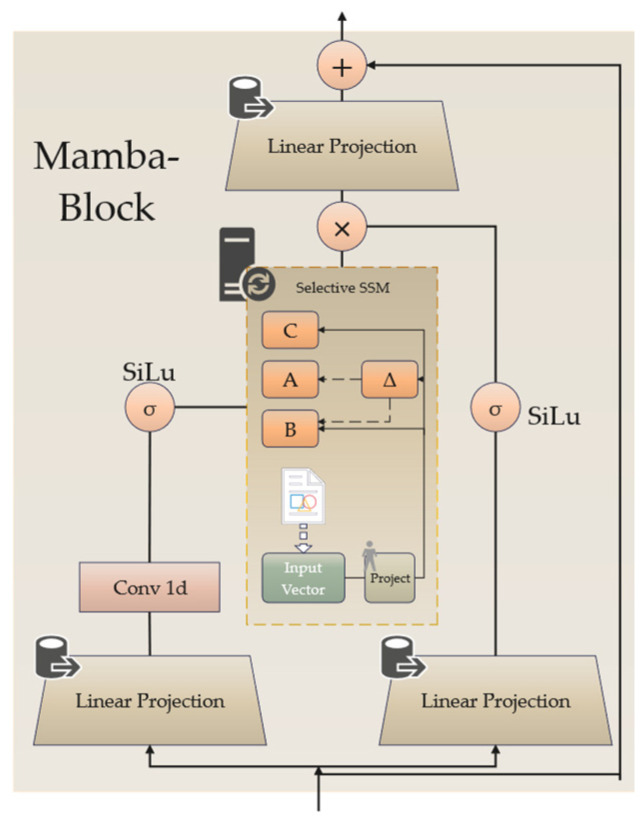
Structural diagram of the Mamba-Block.

The Mamba-Block within the NIDS-Mamba architecture is illustrated in Figure 5. This layer selects relevant data from the input vector and learns time-varying dependencies through an SSM. The internal architecture of the Mamba-Block adopts a gated dual-path design to synergistically process network traffic features. Upon entering the block, the input sequence is bifurcated into two symmetric branches through linear projections. The primary branch (left) first utilizes a 1D convolution layer to aggregate local spatial–temporal correlations within the flow, which is then fed into the Selective SSM module. This module acts as the core engine, performing a hardware-aware selective scan to model long-range dependencies by dynamically adjusting its state transition parameters based on the input content. Simultaneously, the auxiliary branch (right) serves as a gating mechanism, where the projected features are activated by a Sigmoid Linear Unit (SiLU) function to generate a modulation signal. The interaction between these two paths is realized through a Hadamard product, allowing the model to selectively amplify critical attack-related features while suppressing background network noise. Finally, the fused information is projected back to the original dimension and integrated with the initial input via a residual connection, ensuring robust gradient flow and information stability across deep layers.

For the *l*-th NIDS-Mamba block, the input flow vector is defined as $X_{l-1}\in R^{B\times L\times D}$, where *B* is the batch size, *L* represents the sequence length of the network flow, and *D* denotes the feature dimension. To capture high-order dependencies, the input is first mapped to a high-dimensional space via two parallel linear projection functions:

(7) $$x={\mathrm{Linear}}_{x}\left(X_{l-1}\right)\;z={\mathrm{Linear}}_{z}\left(X_{l-1}\right)$$

where $x,z\in R^{B\times L\times \left(E\cdot D\right)}$ serve as the intermediate feature branch and the gating branch, respectively, with *E* being the expansion factor. In the Mamba block, factor *E* is set to 2, effectively projecting the input to a 256-dimensional space. The 1D convolution layer employs a kernel size of 4 to capture local dependencies within the expanded flow features. Subsequently, non-linear transformations are applied to these branches to extract local context:

(8) $$x^{\prime }=\mathrm{SiLu}\left(\mathrm{Conv}1D\left(x\right)\right)\;z^{\prime }=\mathrm{SiLu}\left(z\right)$$

In this formulation, $x^{\prime }\in R^{B\times L\times \left(E\cdot D\right)}$ represents the feature tensor processed by a one-dimensional convolution (Conv1D) and the SiLU activation function. The gated tensor $z^{\prime }\in R^{B\times L\times \left(E\cdot D\right)}$ is used to modulate the information flow. Based on the selective mechanism, the parameter matrices *B*, *C*, and the step size Δ of the SSM are dynamically derived from *x*′ as follows [32]:

(9) $$B=S_{B}\left(x^{\prime }\right)\;C=S_{B}\left(x^{\prime }\right)\;\Delta =\log\left(1+\exp\left(S_{\Delta }\left(x^{\prime }\right)\right)\right)$$

where $S_{B},\;S_{C},$ and $S_{\Delta }$ represent learnable linear transformations. By employing the computed step size Δ, the continuous system matrices *A* and *B* are discretized into $\bar{A}$ and $\bar{B}$:

(10) $$\bar{A}=\exp(\Delta A)\;\bar{B}={\left(\Delta A\right)}^{-1}(\exp(\Delta A)-I)\cdot \Delta B$$

Following the selective SSM scan, the latent output $y\in R^{B\times L\times \left(E\cdot D\right)}$ is generated:

(11) $$y=\mathrm{SelectiveSSM}\left(\bar{A},\;\bar{B},\;C\right)\left(x^{\prime }\right)$$

As illustrated in Equation (12), the output *y* is multiplied by the gated branch *z*′ via the Hadamard product ⊙ and undergoes a final linear projection to revert to the original dimension *D*. This result is combined with the initial input via a residual connection to produce the final block output $X_{l}\in R^{B\times L\times D}$:

(12) $$X_{l}={\mathrm{Linear}}_{o}\left(y⊙z^{\prime }\right)+X_{l-1}$$

b. Self-Attention Layer

**Figure 6:**
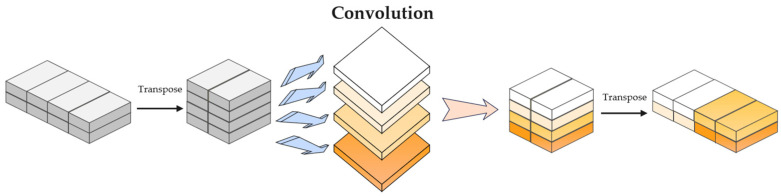
Schematic diagram of the attention mechanism.

The attention mechanism enables each time step to interact with all information across other time steps, thereby capturing long-term dependencies within the traffic sequence. This process is typically implemented utilizing the Scaled Dot-Product [33], as detailed in Figure 6.

Each position of the input $X_{t}$ is multiplied by the weight matrices $W_{Q}$, $W_{K}$, and $W_{V}$ to generate three vectors: Query (Q), Key (K), and Value (V). For each position’s Query, a dot product is computed with the Key of other positions to yield attention scores, which are subsequently normalized via a Softmax function to determine the attention weights for each position. These weights are then multiplied by the corresponding Value to derive the output for each position. Finally, a FFN applies an independent transformation to the output of each position. The specific computational procedure is expressed in Equation (13):

(13) $$Q=X_{t}W_{Q},K=X_{t}W_{K},V=X_{t}W_{V}\;\mathrm{Attention}\left(Q,K,V\right)=\mathrm{Softmax}\left(\frac{QK^{T}}{\sqrt{d_{k}}}\right)V\;X_{\mathrm{att}}=\mathrm{FeedForward}\left(\mathrm{Attention}\left(Q,K,V\right)\right)$$

In the equation above, $d_{k}$ represents the dimension of the Key vector, which is utilized to scale the attention scores.

However, in real-world scenarios, data flows frequently exhibit a substantial amount of homologous communication features, yet traditional self-attention mechanisms compute their similarities regardless. In NIDS-Mamba, not all feature units are required to participate in attention interactions; flow features possessing similar statistical properties should instead be aggregated into a higher-order traffic pattern representation. Directly binding the original input dimensions to the dimensions of the Q, K, and V vectors not only introduces redundant computational overhead but also constrains the model’s learning capacity.

To address these limitations, this section proposes a scalable self-attention mechanism. By introducing two scaling factors, $s_{n}$ and $s_{c}$, it decouples the dimensionality of the input vector from that of the Q, K, and V vectors. The selection of scaling factors $s_{n}$ and $s_{c}$ is determined by the specific resource constraints of the target IoT edge device and the complexity of the network traffic features. $s_{n}$ controls the compression ratio of the temporal dimension (sequence length), while $s_{c}$ governs the dimensionality reduction of the feature space. We employ a multi-head scalable attention mechanism with eight attention heads. By setting the scaling factors $s_{n}$ = 4 and $s_{c}$ = 1, the model effectively aggregates temporal patterns across every four tokens, reducing the computational complexity from quadratic to linear $O\left(L\cdot s_{n}\right)$. In our experiments, these values are optimized through a grid search: a higher scaling factor preserves more fine-grained information but increases computational latency, whereas a lower factor enhances throughput at the cost of potential accuracy degradation. This flexibility allows NIDS-Mamba to be reconfigured for different hardware tiers, from high-performance gateways to low-power sensor nodes. Furthermore, three transformation functions—$f_{q}\left(\cdot \right)$, $f_{k}\left(\cdot \right)$, and $f_{v}\left(\cdot \right)$—are employed to reduce the dimensionality of the weight matrices $W_{Q}$, $W_{K}$, and $W_{V}$. To implement the transformation functions, we employ a combination of depthwise separable convolution and linear projection. For an input sequence $X\in R^{L\times D}$, the transformation is defined as follows:

(14) $$f\left(X\right)=\mathrm{Linear}\left(\mathrm{DWConv}\left(X\right)\right)$$

where DWConv denotes a depthwise convolution with a stride corresponding to the scaling factor $s_{n}$, effectively aggregating neighboring flow features into a higher-order representation. The Linear layer then projects the hidden dimension *D* to the target scale $s_{c}$. By utilizing depthwise convolution, we maintain a minimal parameter count while capturing local spatial–temporal correlations that standard point-wise projections would miss. The detailed calculation is presented in Equation (15):

(15) $$\left\{\begin{array}{c} Q^{\prime }=f_{q}\left(X_{t}\right)W_{Q}^{\prime }\\ K^{\prime }=f_{k}\left(X_{t}\right)W_{K}^{\prime }\\ V\prime =f_{v}\left(X_{t}\right)W_{V}^{\prime } \end{array}\right.\;\mathrm{Attention}\left(Q^{\prime },K^{\prime },V^{\prime }\right)=\mathrm{Softmax}\left(\frac{Q^{\prime }{K^{\prime }}^{T}}{\sqrt{d_{k}}}\right)V^{\prime }$$

where $W_{Q}^{\prime }\in R^{D\times s_{n}}$, $W_{K}^{\prime }\in R^{D\times s_{n}}$, and $W_{V}^{\prime }\in R^{D\times s_{n}}$. The dimensional scaling of the three weight matrices is achieved through $f_{q}\left(\cdot \right)$, $f_{k}\left(\cdot \right)$, and $f_{v}\left(\cdot \right)$, a procedure that effectively mitigates unnecessary intermediate multiplications. In practice, the implementation of these three transformation functions relies on the combined synergistic effects of convolution and linear projection. The scalable self-attention mechanism preserves the dimensions of the input matrix, ensuring rigorous dimensional alignment between the input and output.

Unlike the standard self-attention mechanism, which suffers from $O\left(L^{2}\right)$ complexity, the proposed scalable mechanism reduces the complexity to $O\left(L\cdot s_{n}\right)$. While existing efficient attention variants, such as Linformer or Performer, often use random projections or low-rank approximations, our approach specifically leverages the redundancy in NetFlow data through learnable convolutional kernels. This ensures that the model does not just mathematically compress the input, but actively filters homologous communication patterns common in IoT botnet attacks, thereby improving the recall of stealthy anomalies.

c. Convolution Layer

**Figure 7:**
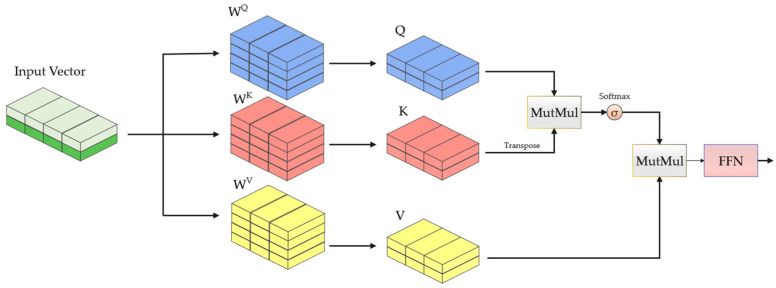
Schematic diagram of the convolution process.

The function of the convolution layer is to expand the model’s receptive field, enhancing its capability to model the interactive correlations of both local and global information within the flow sequence. Throughout this procedure, the dimensions of the input and output sequences remain consistent. The convolution process is illustrated in Figure 7.

The dedicated convolution layer following the FFN utilizes a 3×1 kernel with a stride of 1. This configuration expands the receptive field to integrate global spatio-temporal contexts without altering the sequence dimensionality.

#### 2.3.3. MLP Head

Because NIDS-Mamba operates as a sequence-to-sequence model, its sequential output must be explicitly converted into NIDS classification results. Although all outputs from NIDS-Mamba could theoretically be fed directly into a MLP, doing so would cause the parameter count to escalate exponentially alongside sequence length. Given that NIDS classification tasks predominantly involve only the category of the final flow (e.g., distinguishing between benign and attack traffic), the final context embedding vector is directly utilized as the input to the classification head to significantly reduce computational complexity.

**Figure 8:**
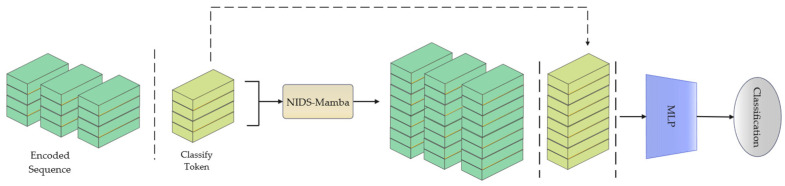
Schematic diagram of the MLP Head input process.

As illustrated in Figure 8, the MLP Head operates by selectively extracting the high-dimensional hidden state corresponding to the Classify Token appended at the sequence terminus. Since the NIDS-Mamba backbone preserves the sequential dimensionality throughout its four layers, this specific vector at the final index serves as a global feature descriptor that aggregates the spatio-temporal context of the entire network flow. By isolating this representative embedding for classification rather than flattening the entire output sequence, the model effectively decouples the parameter complexity of the MLP from the sequence length *L*. This architectural choice allows the system to maintain a constant computational footprint in the decision-making stage, which is particularly advantageous for resource-constrained IoT gateways. The extracted vector is then processed through fully connected layers to map the learned representations into a probability distribution, facilitating the final distinction between benign traffic and various attack categories such as DoS/DDoS or Web Attacks.

## 3. Experimental Results and Analysis

### 3.1. Experimental Setup

To ensure the reproducibility of the results, all experiments were conducted on a high-performance workstation equipped with an Intel Core i9-13900K CPU, 64 GB of RAM, and an NVIDIA GeForce RTX 4090 GPU (24 GB VRAM). The software environment was built on Ubuntu 22.04 LTS using the PyTorch 2.1 framework with CUDA 12.1 acceleration.

### 3.2. Introduction to Traffic Datasets

The datasets utilized in this experiment are standard NIDS feature sets based on the NetFlow network metadata collection protocol and system, specifically NF-UNSW-NB15 and NF-CSE-CIC-IDS2018 [12,34,35]. These two datasets are subsequently employed to validate the effectiveness of the proposed model. A critical issue inherent in the baseline forms of these datasets, namely UNSW-NB15 and CSE-CIC-IDS2018, is the lack of a standardized feature set. Because each publicly available dataset utilizes a unique set of proprietary features, it becomes exceedingly difficult to compare the performance of machine learning-based traffic classification models across different datasets, thereby hindering the evaluation of these systems’ generalization capabilities across diverse network scenarios.

**Figure 9:**
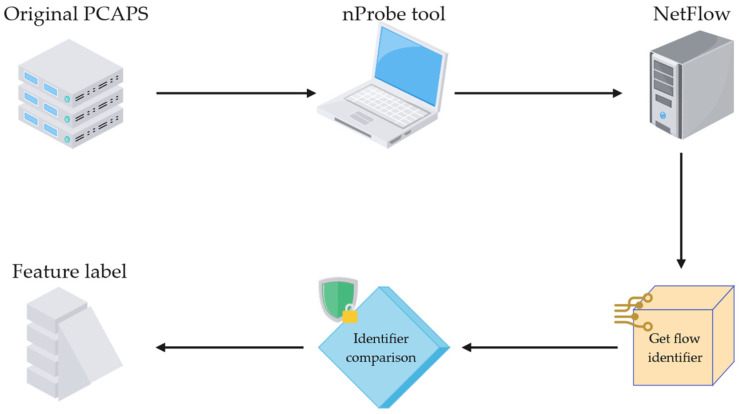
Schematic diagram of the dataset standardization process.

To overcome the limitations, this study utilizes NetFlow to standardize the features of the benchmark datasets [8]. Figure 9 illustrates the processing pipeline from the raw datasets to the standardized datasets.

The nProbe tool is employed to extract 43 NetFlow features from publicly available pcap files. The output format is designated as a text flow, where each feature is separated by a comma (,) to facilitate its use as a CSV file. By matching five flow identifiers (source/destination IP, port, and protocol) with the ground-truth attack events published in the original datasets, two label features are generated. If a data flow falls within an attack event, it is labeled as an attack flow, and its corresponding attack type is recorded in the attack label; otherwise, the sample is labeled as a benign flow. The standardized flow features are presented in Table 2 below.

The 43 standardized features mentioned above represent the temporal and statistical behavior of network flows, enabling the NIDS-Mamba model to identify complex attack patterns without inspecting individual packet payloads.

**Overview of NF-CSE-CIC-IDS2018:** In 2018, the Communications Security Establishment (CSE) and the Canadian Institute for Cybersecurity (CIC) collaboratively released the CSE-CIC-IDS2018 dataset [12,34]. Benign packets were captured during normal network scenarios, while attack scenarios were executed by one or multiple machines outside the target network. Following NetFlow standardization, the total number of data flows is 18,893,708, comprising 16,635,567 (88.05%) benign samples and 2,258,141 (11.95%) attack samples. Table 3 details the distribution of all flows within the NF-CSE-CIC-IDS2018 dataset. We obtained this dataset on the webpage (https://www.unb.ca/cic/datasets/ids-2018.html, accessed on 11 November 2025).

**Overview of NF-UNSW-NB15:** The cyber range laboratory of the Australian Centre for Cyber Security (ACCS) released the widely used UNSW-NB15 dataset in 2015 [12,35]. The raw network packets of the dataset were generated using the IXIA PerfectStorm tool within the institution’s cyber range, which is capable of synthesizing a hybrid dataset of contemporary normal activities and synthetic contemporary attack behaviors. Following NetFlow standardization, the total number of data flows is 2,390,275, consisting of 2,295,222 (96.02%) benign samples and 95,053 (3.98%) attack samples. The attack samples are further subdivided into nine categories. Table 4 presents the distribution of all flows within the NF-UNSW-NB15 dataset. We obtained this dataset from the webpage (https://staff.itee.uq.edu.au/marius/NIDS_datasets/, accessed on 11 November 2025).

### 3.3. Evaluation Metrics

To rigorously evaluate the proposed model against the challenges identified in the introduction, our performance metrics are categorized into two dimensions: (1) Detection Robustness, which includes accuracy, F1-score, G-Mean, MCC, and AUC to ensure high-performance anomaly identification under class imbalance; and (2) System Efficiency, which assesses throughput (flows/s), parameter scale (Storage), and peak memory usage (RAM) to verify its suitability for resource-constrained IoT environments.

Given the extreme class imbalance in realistic NIDS datasets (e.g., attack samples constitute only 3.98% in NF-UNSW-NB15), relying solely on accuracy can be misleading. Therefore, this study incorporates AUC, G-Mean, and MCC to robustly evaluate the model’s performance on minority classes. As a threshold-independent metric, AUC is particularly suitable for the imbalanced classification problem addressed in this study. It evaluates the model’s discriminative power across all possible classification thresholds, providing a more comprehensive assessment of its ability to distinguish between benign and malicious traffic without being biased by class distribution. G-Mean measures the balance between majority and minority class classification accuracies, while MCC provides a reliable statistical rate that produces a high score only if the prediction obtained good results in all four of the confusion matrix categories.

### 3.4. Experimental Process and Results Analysis

#### 3.4.1. Binary Classification Experiment

This section validates the effectiveness of the proposed NIDS-Mamba model on the NF-UNSW-NB15 and NF-CSE-CIC-IDS2018 datasets, comparing its detection performance with various intrusion detection models. These include the classical machine learning model Random Forest (RF) and representative models in the intrusion detection domain, such as BiLSTM and CNN-BiLSTM [11,36]. BERT is selected as a comparative model because it is fundamentally constructed from multiple Transformer encoder blocks, allowing for a comparison of performance differences between the Transformer and Mamba architectures in the intrusion detection domain. Furthermore, traffic structures closely resemble natural language, a domain where BERT excels. To enhance experimental efficiency, a pre-trained lightweight BERT is fine-tuned for this experiment. The experimental results on both datasets are presented in Table 5 and Table 6 below.

As indicated by the experimental results in the aforementioned tables, on the NF-UNSW-NB15 dataset, the detection accuracy of the NIDS-Mamba model improves by 3.75% compared to RF, 2.56% compared to BiLSTM, 2.37% compared to CNN-BiLSTM, and 6.47% compared to BERT. The proposed model also exhibits a certain degree of improvement in precision and F1-score relative to the other models. Although its recall is slightly lower than that of BERT, BERT tends to misclassify an excessive amount of normal traffic as anomalous, resulting in exceedingly low scores across all other metrics. On the NF-CSE-CIC-IDS2018 dataset, the NIDS-Mamba model achieves the optimal detection performance, with an accuracy, precision, recall, FPR, and F1-score of 98.32%, 97.43%, 96.53%, 1.26%, and 96.98%, respectively.

**Figure 10:**
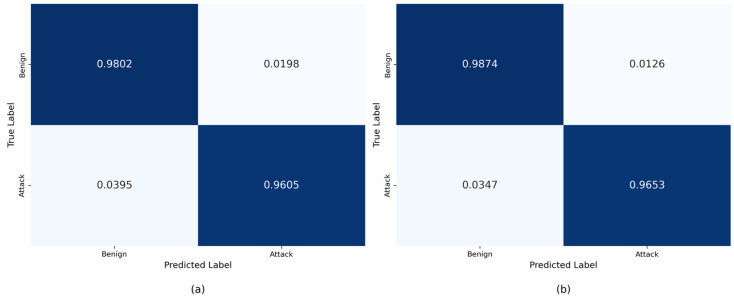
Confusion matrix of the binary classification experiment: (**a**) NF-UNSW-NB15; (**b**) NF-CSE-CIC-IDS2018.

To further examine the classification performance of NIDS-Mamba at a granular level, Figure 10 presents the normalized confusion matrices for both datasets. The diagonal elements represent the percentage of correctly classified instances for benign and malicious flows. It is evident that NIDS-Mamba maintains a high true positive rate (TPR) even in the NF-UNSW-NB15 dataset, where attack samples are extremely sparse. Notably, compared to the BERT model, which suffers from excessive false alarms, NIDS-Mamba significantly reduces the FPR, as reflected by the minimal values in the bottom-left quadrant of the matrices. This visualization reinforces the robust discriminative power of the proposed Mamba-based architecture in handling long-tail distributions.

Notably, while all models exhibit high accuracy, the superior performance of NIDS-Mamba is most evident in the MCC metric. On the highly imbalanced NF-UNSW-NB15 dataset, NIDS-Mamba achieves an MCC of 0.7915, significantly outperforming BERT and LSTM variants. This robust MCC and G-Mean indicate that the proposed Mamba architecture does not achieve high accuracy merely by voting for the majority benign class, but genuinely learns the discriminative features of minority attack patterns. The superior performance in MCC and AUC, especially on the highly imbalanced NF-UNSW-NB15 dataset, is intrinsically linked to the selective scan mechanism of the Mamba block. Unlike BERT, which may suffer from “attention dispersion” in noisy network traffic, NIDS-Mamba’s content-aware filtering allows the model to prioritize rare attack-related temporal features while suppressing redundant background noise. This ensures that the high accuracy is derived from genuine discriminative learning of minority patterns rather than a biased preference for the majority benign class.

Furthermore, the NIDS-Mamba model demonstrates exceptional performance in terms of the AUC metric. On the NF-UNSW-NB15 dataset, it achieves an AUC of 0.9983, which is higher than RF, BiLSTM, and significantly outperforms the BERT model. A similar trend is observed on the NF-CSE-CIC-IDS2018 dataset, where NIDS-Mamba reaches a near-perfect AUC of 0.9996. As a threshold-independent metric, these superior AUC values confirm that the proposed architecture maintains a high true positive rate and a low false positive rate across all possible classification thresholds. This indicates that the model has learned highly discriminative feature representations, providing robust and stable detection capabilities even when dealing with the high-dimensional and non-linear patterns characteristic of network intrusions.

The experimental results from both datasets reveal that all models experience a certain degree of performance degradation on NF-UNSW-NB15 compared to NF-CSE-CIC-IDS2018. This decline is attributed to the extreme class imbalance in the former, which exhibits a pronounced long-tail distribution where the sample size of certain malicious traffic categories is less than 0.1% of the normal traffic. Consequently, the classification capability for low-frequency attack types is significantly diminished. However, NIDS-Mamba effectively mitigates the issue of class imbalance better than the other models, maintaining stability across various metrics, thereby demonstrating the effectiveness of the proposed model architecture in the field of network intrusion detection.

#### 3.4.2. Training and Inference Efficiency

The objective of this experiment is to evaluate the training and inference efficiency of NIDS-Mamba. Training time can be measured by timing each batch, dividing this time by the batch size, and averaging it across all batches utilized during the training process. To evaluate inference time, the experiment initially records the inference time for a single batch from start to finish. To ensure result stability and preclude caching effects, four repeated measurements are conducted on the same data batch to establish a reliable time range. Subsequently, the same measurement process is repeated for 50 randomly selected batches, and the inference times of these batches are averaged. The final results are expressed as the traffic volume the model can ingest per second (flows/s). The experimental results are presented in Table 7 below.

Among the models evaluated in the experiment, the throughput during training and inference, from highest to lowest, is NIDS-Mamba, CNN-BiLSTM, and BERT. From the perspective of model architecture, the excessively large parameter count of BERT leads to substantial computational overhead and high GPU memory utilization during training and inference, thereby constraining its throughput. Due to its recurrent architecture, CNN-BiLSTM exhibits relatively weak parallelization capabilities, which consequently restricts its training and inference speeds. In contrast, NIDS-Mamba employs a selective scanning mechanism rather than convolution for recurrent model computations, which, combined with parallel computing capabilities, substantially accelerates computation speeds on the GPU. In real-world network environments, the stringent requirement for high real-time processing efficiency in traffic detection models further underscores the practical value of the proposed model.

The dramatic improvement in inference throughput stems from the parallelizable hardware-aware scan that replaces the sequential processing found in traditional recurrent architectures like BiLSTM. While Transformers achieve parallelism at the cost of quadratic complexity $O\left(L^{2}\right)$, NIDS-Mamba maintains a linear-time complexity $O\left(L\cdot s_{n}\right)$. This allows the model to exploit GPU acceleration more effectively during inference, bridging the gap between high-level modeling and the real-time requirements of IoT gateways.

To further evaluate the model’s suitability for resource-constrained environments, we compared the parameter scale and peak memory consumption during inference. After conducting comparative experiments, it was found that NIDS-Mamba achieves a significant reduction in model size compared to CNN-BiLSTM and BERT-based variants. Our model requires only 1.12 M parameters, which is approximately 1/10th the size of a standard lightweight BERT model used in NIDS. Furthermore, the peak RAM usage during a single flow inference is recorded at 5.4 MB, demonstrating its compatibility with the memory limits typical of embedded IoT gateways and industrial edge controllers. While direct energy measurements were not conducted in this study, the model’s energy efficiency can be logically inferred from its high throughput and compact footprint. Energy consumption is defined as the product of power and execution time (E=P×t); NIDS-Mamba’s superior inference speed means the processor remains in a high-power state for a significantly shorter duration per flow compared to Transformer or BiLSTM baselines. Furthermore, the compact 5.4 MB peak RAM footprint facilitates the model’s execution within the internal SRAM of many embedded platforms, thereby minimizing the energy overhead typically associated with external memory (e.g., DRAM) accesses.

It should be noted that we conducted the core evaluation in a GPU-accelerated environment to establish performance benchmarks. The architectural parameters were specially adjusted to reflect the limitations of the heterogeneous IoT hierarchy. This achieved hardware efficiency, characterized by the low-megabyte memory demand and million-level parameter scale, is specifically tailored to bridge the gap between high-performance edge gateways and industrial IoT edge nodes with limited dedicated memory. The linear complexity $O\left(L\cdot s_{n}\right)$ ensures that the computational demand scales efficiently across these devices without causing the “memory wall” typically seen in Transformer-based NIDS.

#### 3.4.3. Multiclass Classification Experiment

**Figure 11:**
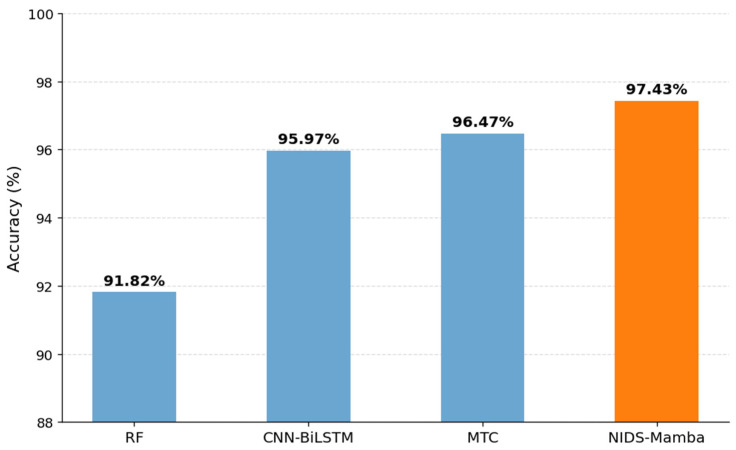
Results of multiclass detection accuracy.

This experiment aims to verify the identification efficacy of the proposed model against various types of network attacks. Given the minuscule proportion of attack samples in the NF-UNSW-NB15 dataset, this experiment validates the multiclass classification performance of different models exclusively on the NF-CSE-CIC-IDS2018 dataset. Because BERT underperformed to achieve satisfactory classification results in the binary classification experiment, 1DCNN-Transformer (MTC) is selected as an alternative comparative model [37]. This architecture also incorporates the Transformer but features significantly fewer encoder layers than BERT. Additionally, the comparative models included in this section are RF and CNN-BiLSTM. The overall detection accuracy of the different models is illustrated in Figure 11.

The detection results indicate that the multiclass detection accuracy of NIDS-Mamba is 97.43%, achieving the best performance among the compared models. It is also noteworthy that the detection accuracy of MTC is 97.12%, which is marginally inferior to NIDS-Mamba but superior to CNN-BiLSTM (96.58%) and RF (95.25%). This suggests that in the NIDS domain, a shallow Transformer architecture outperforms the deep BERT model. Unlike traditional RNNs and their variants, both the proposed method and MTC leverage attention mechanisms to extract features from sequential data, yielding robust overall detection performance in the multiclass flow identification task.

**Figure 12:**
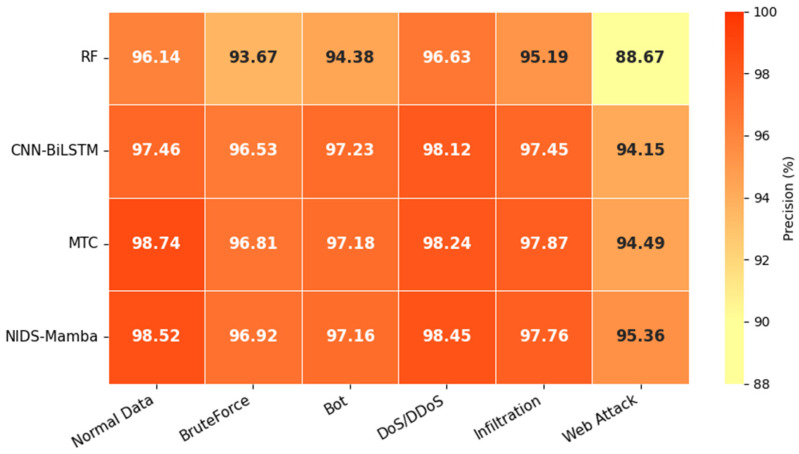
Results of multiclass detection precision.

Figure 12 presents the multiclass detection precision of the different models. As the figure demonstrates, NIDS-Mamba attains the highest detection precision for three attack types: BruteForce, DoS/DDoS, and Web Attack, at 96.92%, 98.45%, and 95.36%, respectively. Regarding the detection precision for normal traffic, it is 0.22% lower than MTC; for Bot, it is 0.07% lower than CNN-BiLSTM; and for Infiltration, it is 0.11% lower than MTC. Although it does not achieve the highest precision across all attack categories, the margin of difference compared to other detection models is negligible. Therefore, from an overall perspective, NIDS-Mamba exhibits excellent detection precision across various traffic types.

**Figure 13:**
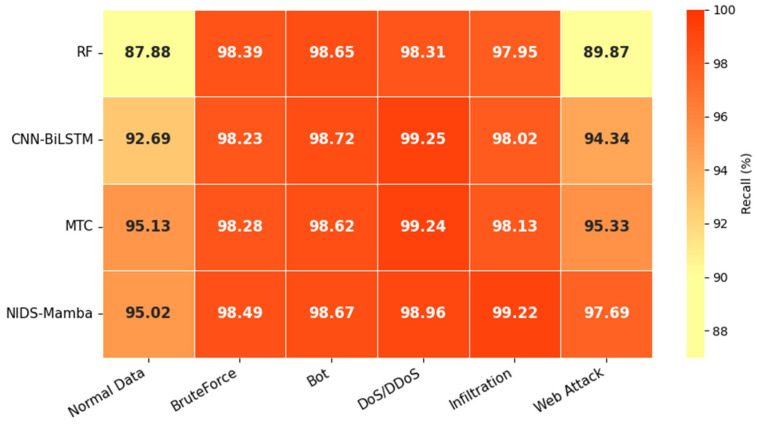
Results of multiclass detection recall.

The multiclass detection recall rates of the different models are illustrated in Figure 13. As shown, NIDS-Mamba achieves the highest detection recall for BruteForce, Infiltration, and Web Attack, at 98.49%, 99.22%, and 97.69%, respectively. Notably, the recall rate for Web Attack is improved by at least 2.36%. Given that this class of samples constitutes the smallest proportion of the dataset, this demonstrates that NIDS-Mamba not only maintains high recall for the majority attack classes but also possesses strong detection capabilities for minority attack classes.

The significant recall gains for stealthy, low-frequency attacks such as “Web Attack” are attributed to the synergistic interaction between the Mamba block and the dynamic sparse attention layer. While the Mamba block performs long-range sequence filtering with linear efficiency, the attention mechanism recovers fine-grained global dependencies that might otherwise be lost during the state compression of a pure SSM. This hybrid structure ensures that even subtle, multi-stage attack patterns are captured without being overshadowed by dominant traffic flows.

**Figure 14:**
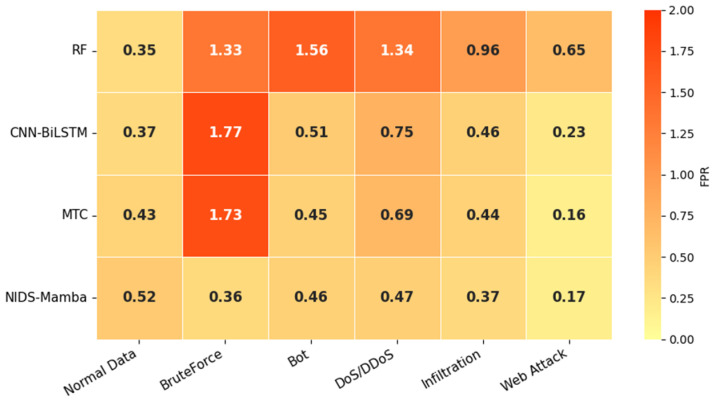
Results of multiclass detection FPR.

Figure 14 illustrates the multiclass detection FPR of the different models. NIDS-Mamba exhibits the lowest FPR on BruteForce, DoS/DDoS, and Infiltration attack types, at 0.36%, 0.47%, and 0.37%, respectively. The detection FPR remains stable and below 1% across all attack categories, demonstrating that even under the premise of significant class imbalance within the flow dataset, the proposed model can consistently maintain a low false positive rate.

**Figure 15:**
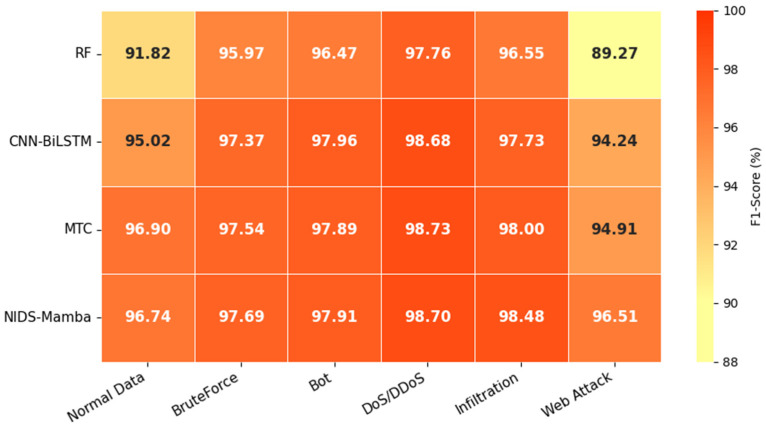
Results of multiclass detection F1-score.

The multiclass F1-scores of the different models on the NF-CSE-CIC-IDS2018 dataset are presented in Figure 15. NIDS-Mamba yields F1-scores of 96.74%, 97.69%, 97.91%, 98.70%, 98.48%, and 96.51% for the normal traffic category, BruteForce, Bot, DoS/DDoS, Infiltration, and Web Attack categories, respectively. It achieves the best F1-scores on BruteForce, DoS/DDoS, Infiltration, and Web Attack. In summary, NIDS-Mamba can more effectively model the relationships among multidimensional features when addressing network intrusion detection tasks, particularly in scenarios characterized by data imbalance and complex attack patterns. Compared to the Transformer-based MTC method, the proposed method demonstrates enhanced adaptability and stability when detecting small-sample or highly stealthy attack types.

## 4. Discussion

### 4.1. Research Findings

In this paper, we address fundamental challenges faced by NIDSs in large-scale and resource-constrained network environments, particularly those involving IoT and sensor networks. By introducing the NIDS-Mamba architecture, we aim to achieve both high detection accuracy and efficient real-time processing, which are critical requirements for edge computing.

Experimental results demonstrate that NIDS-Mamba achieves outstanding detection performance. For instance, on the NF-CSE-CIC-IDS2018 dataset, the model attains an accuracy of 98.32% for binary classification and 97.43% for multiclass classification. More importantly, for minority attack types such as Web Attack, the recall rate improves by at least 2.36% compared to baseline models. This is particularly significant in IoT scenarios, where attacks such as botnet propagation (e.g., Mirai-like behaviors), reconnaissance scanning, and low-frequency infiltration events typically constitute only a small fraction of overall traffic. Crucially, the model yields exceptional AUC scores of 0.9983 and 0.9996 on the NF-UNSW-NB15 and NF-CSE-CIC-IDS2018 datasets, respectively. The strong MCC, G-Mean, and AUC performance of NIDS-Mamba indicates that the model effectively captures these rare but critical attack patterns without being biased toward dominant benign traffic. The high AUC values further confirm that NIDS-Mamba maintains superior discriminative power across all potential decision thresholds, making it highly suitable for real-world IoT threat environments.

In addition to accuracy, the model demonstrates substantial improvements in throughput. NIDS-Mamba achieves an inference throughput of approximately 7533–8463 flows per second, significantly outperforming Transformer-based models such as BERT. In practical IoT deployments, edge gateways typically handle traffic from tens to thousands of connected devices, with flow rates generally well below this threshold. Therefore, the achieved throughput indicates that NIDS-Mamba is capable of meeting real-time detection requirements in typical IoT gateway scenarios, while still maintaining sufficient computational headroom for additional edge tasks.

Furthermore, the linear computational complexity of the Mamba-based architecture enables efficient utilization of hardware resources, reducing both latency and energy consumption. This characteristic is particularly valuable for edge environments, where power efficiency and sustained operation are critical constraints.

### 4.2. Comparative Analysis with Related Work

Existing research on network intrusion detection has primarily evolved along two directions: recurrent neural networks (RNNs/LSTMs) and Transformer-based architectures. While LSTM-based models can capture temporal dependencies, their sequential nature limits parallelization and reduces throughput, making them less suitable for high-speed or real-time IoT environments. Transformer-based models improve dependency modeling through attention mechanisms but introduce substantial computational and memory overhead due to their quadratic complexity, which restricts their applicability in resource-constrained edge systems.

Recent specialized architectures, such as GNN-based and TCN-based NIDS, provide localized or topological advantages that complement our SSM approach. GNNs excel at detecting coordinated multi-stage attacks by analyzing the connectivity patterns of IP nodes [22]; however, the high computational cost of graph construction and neighborhood aggregation often precludes their use in resource-constrained IoT gateways. TCNs, while efficient in capturing local temporal patterns via fixed-size receptive fields, may lack the dynamic long-range filtering capabilities inherent in Mamba’s selective mechanism [38]. NIDS-Mamba bridges this gap by integrating a lightweight state space with dynamic sparse attention, offering a global receptive field similar to GNNs but with the linear complexity of advanced TCNs. This hybrid design ensures that stealthy, long-tail attack patterns—often missed by fixed-window convolutional models—are effectively captured without the memory explosion typical of graph-based or standard Transformer architectures.

In addition, NIDS-Mamba achieves a more favorable balance between detection performance and computational efficiency by leveraging a hybrid architecture that integrates selective state space modeling with attention mechanisms. While pure Mamba models achieve near-linear complexity, they inherently face an “information bottleneck” where the fixed-size recurrent state must act as a compressed summary of all past inputs, which may struggle to preserve the fine-grained historical details necessary for identifying sophisticated or stealthy attacks. By incorporating a dynamic attention mechanism, our architecture provides a global receptive field to compensate for Mamba’s state compression, capturing critical dependencies and multidimensional feature interactions that a pure SSM might overlook. This synergy enables both high throughput and superior modeling capability without incurring the prohibitive costs associated with standard Transformers.

In addition, recent studies have explored lightweight or IoT-oriented NIDS solutions, often based on simplified CNNs, shallow RNNs, or feature-engineered machine learning models. While these approaches reduce computational overhead, they typically sacrifice the ability to model long-range temporal dependencies or complex attack behaviors. In contrast, the proposed NIDS-Mamba architecture maintains strong expressive power while significantly improving efficiency, thereby bridging the gap between lightweight IoT-compatible models and high-performance deep learning-based NIDSs. By exploiting the complementary expressive power of SSMs and attention, NIDS-Mamba ensures high-fidelity detection across diverse and resource-constrained network scenarios.

### 4.3. Identified Limitations

Despite the promising results, several limitations remain:

(1) **Hardware-specific validation and energy quantification**
  A primary limitation is that the current performance evaluation was conducted in high-performance GPU environments rather than on physical resource-constrained edge devices. While workstation benchmarks provide a stable baseline for architectural efficiency, they do not fully reflect the complexities of real-world heterogeneous IoT hardware. Specifically, this paper lacks direct energy consumption metrics for specific low-power hardware targets. Furthermore, although the peak RAM and parameter footprint suggest compatibility with embedded systems, the absence of on-chip validation means that hardware-specific constraints—such as memory latency and cache misses on ARM Cortex-M microcontrollers—remain unquantified.
(2) **Sensitivity to Long-tail Attack Distributions**
  Although SMOTE oversampling alleviates class imbalance, detection sensitivity still degrades for extremely rare and stealthy attacks that constitute less than 0.1% of total traffic. Oversampling techniques alone may introduce statistical noise or lead to overfitting on minority classes, potentially overshadowing the subtle characteristics of sophisticated, multi-stage intrusions.
(3) **Limited adaptability to dynamic threat environments**
  The current model relies on offline supervised training using labeled datasets. In dynamic IoT environments, where new attack patterns emerge continuously, this may limit real time adaptability. Consequently, the model may struggle to identify evolving, zero-day attack patterns in real time without periodic retraining on updated datasets.

### 4.4. Future Work

To overcome the aforementioned limitations, future research might take the following directions:

(1) **Edge deployment and model compression**
  A key direction is enabling the efficient deployment of NIDS-Mamba on real-world edge hardware platforms. This includes the application of model compression techniques such as knowledge distillation, quantization, and structured pruning to reduce computational overhead and memory footprint [39,40]. Future implementations will target representative edge devices, including embedded AI platforms (e.g., NVIDIA Jetson), single-board computers (e.g., Raspberry Pi), and low-power microcontroller units (e.g., ARM Cortex-M series). Achieving efficient inference on such devices will be critical for practical IoT security applications.
  To bridge the gap between high-level modeling and practical deployment, future work will implement hardware-specific optimizations, including 8-bit quantization and structured pruning. We plan to deploy NIDS-Mamba on a hierarchy of IoT devices, ranging from Raspberry Pi 4B to ARM Cortex-M7 microcontrollers. This will enable the measurement of actual energy savings (mJ/flow) using professional power-profiling tools in real-world battery-driven scenarios.
(2) **Enhancing detection of rare and unknown attacks**
  Curriculum Learning can be introduced to guide the model through a progressive training process from easy to difficult samples [41]. Additionally, generative models such as GANs or diffusion models may be employed to synthesize high-fidelity minority attack flows [41], thereby improving the model’s ability to extract representations from small-sample anomalies. We will explore Generative Adversarial Networks (GANs) and Curriculum Learning to synthesize high-fidelity attack samples and guide the model through a progressive training process.
(3) **Improving adaptability via continual and meta-learning**
  To overcome the limitations of static supervised models, NIDS-Mamba may be combined with meta-learning or continual learning mechanisms [9,42]. To shift from static detection to dynamic defense, future research will integrate it to enable NIDS-Mamba to adapt to zero-day threats with minimal labeled data.

## Figures and Tables

**Table 1:** Model hyperparameters.

Configuration	Parameter	Value
Global Settings	Model Layers (*L*)	4
	Hidden Dimension (*D*)	128
	Batch Size	1024
Mamba Block	Expansion Factor (*E*)	2
	SSM State Dimension (*N*)	16
	Conv1D Kernel Size	4
Self-Attention	Number of Heads	8
	Temporal Scale Factor ($s_{n}$)	4
	Channel Scale Factor ($s_{c}$)	1
Convolution Layer	Kernel Size	3
	Dropout Rate	0.1
MLP Head	Hidden Units	[256, 128]

**Table 2:** Standardized NetFlow features.

Standardized Methods	Standard Flow Features
NetFlow	(1) IPV4_SRC_ADDR (2) IPV4_DST_ADDR (3) L4_SRC_PORT (4) PROTOCOL
	(5) L7_PROTO (6) IN_PKTS (7) OUT_PKTS (8) FLOW_DURATION_MILLISE
	(9) TCP_FLAGS (10) CLIENT_TCP_FLAGS (11) SERVER_TCP_FLAGS
	(12) DURATION_IN (13) DURATION_OUT (14) MIN_TTL (15) MAX_TTL
	(16) LONGEST_FLOW_PKT (17) SHORTEST_FLOW_PKT (18) MIN_IP_PKT
	(19) MAX_IP_PKT_LEN (20) SRC_TO_DST_SECOND_BYTES
	(21) DST_TO_SRC_SECOND_BYTES (22) RETRANSMITTED_IN_BYTES
	(23) RETRANSMITTED_IN_PKTS (24) RETRANSMITTED_OUT_BYTES
	(25) RETRANSMITTED_OUT_PKTS (26) L4_DST_PORT (27) IN_BYTES
	(28) OUT_BYTES (29) SRCTO_DST_AVG_THROUGHPUT
	(30) DST_TO_SRC_AVG_THROUGHPUT (31) NUM_PKTS_UP TO_128_BYTES
	(32) NUM_PKTS_128_TO_256 BYTES (33) NUM_PKTS_256_TO_512_BYTES
	(34) NUM_PKTS_512_TO_1024_BYTES (35) NUM_PKTS_1024TO_1514 BYTES
	(36) TCP_WIN_MAX_IN (37) TCP_WIN_MAX_OUT (38) ICMP_TYPE
	(39) ICMP_IPV4_TYPE (40) DNS_QUERY_ID (41) DNS_QUERY_TYPE
	(42) DNS_TTL_ANSWER (43) FTP_COMMAND_RET_CODE

**Table 3:** Flow distribution of the NF-CSE-CIC-IDS2018-v2 dataset.

Data Type	Description	Record the Number of Connections
Normal Data	Benign	16,635,567
Attack Data	Brute Force	120,912
	Bot	143,097
	DoS/DDoS	1,874,269
	Infiltration	116,361
	Web Attack	3502

**Table 4:** Flow distribution of the NF-UNSW-NB15 dataset.

Data Type	Description	Record the Number of Connections
Normal Data	Benign	2,295,222
Attack Data	Fuzzers	22,310
	Analysis	2299
	Backdoor	2169
	DoS	5794
	Exploits	31,551
	Generic	16,560
	Reconnaissance	12,779
	Shellcode	1427
	Worms	164

**Table 5:** Binary classification experimental results for NF-UNSW-NB15.

Model	Accuracy (%)	Precision (%)	Recall (%)	FPR (%)	F1-Score (%)	G-Mean (%)	MCC	AUC
RF	91.68	93.83	94.17	3.56	94.01	95.30	0.6866	0.9754
BiLSTM	92.87	93.45	94.23	3.52	93.83	95.35	0.6890	0.9816
CNN-BiLSTM	93.06	96.21	94.69	3.06	95.44	95.81	0.7160	0.9952
BERT	88.96	81.72	98.86	5.96	89.47	96.42	0.6147	0.9505
**NIDS-Mamba**	**95.43**	**96.42**	96.05	**1.98**	**96.23**	**97.03**	**0.7915**	**0.9983**

The bold numbers in the last row indicate that compared to other models, NIDS-Mamba achieves the best performance in this metric. If not bolded, it means that the performance of other models is higher than that of NIDS-Mamba.

**Table 6:** Binary classification experimental results for NF-CSE-CIC-IDS2018.

Model	Accuracy (%)	Precision (%)	Recall (%)	FPR (%)	F1-Score (%)	G-Mean (%)	MCC	AUC
RF	95.97	96.53	93.48	1.76	94.98	95.83	0.7925	0.9850
BiLSTM	96.39	96.78	93.97	1.78	95.35	96.07	0.7939	0.9896
CNN-BiLSTM	98.16	97.21	94.68	1.65	95.93	96.50	0.8079	0.9972
BERT	97.43	96.01	94.72	3.48	95.36	95.62	0.6938	0.9638
**NIDS-Mamba**	**98.32**	**97.43**	**96.53**	**1.26**	**96.98**	**97.63**	**0.8503**	**0.9996**

The bold numbers in the last row indicate that compared to other models, NIDS-Mamba achieves the best performance in this metric. If not bolded, it means that the performance of other models is higher than that of NIDS-Mamba.

**Table 7:** Results of the model traffic throughput per unit time.

Dataset	Model	Throughput (Flows/s)	
		Train	Inference
NF-UNSW-NB15	CNN-BiLSTM	1090	6327
	BERT	78	659
	**NIDS-Mamba**	**1628**	**7533**
NF-CSE-CIC-IDS2018	CNN-BiLSTM	954	7465
	BERT	76	251
	**NIDS-Mamba**	**1345**	**8463**

The bold numbers in the last row indicate that compared to other models, NIDS-Mamba achieves the best performance in this metric. If not bolded, it means that the performance of other models is higher than that of NIDS-Mamba.

## Data Availability

The datasets analyzed in this study are publicly available. The NF-CSE-CIC-IDS2018 dataset can be found at: https://www.unb.ca/cic/datasets/ids-2018.html (accessed on 15 March 2026). The NF-UNSW-NB15 dataset can be found at: https://staff.itee.uq.edu.au/marius/NIDS_datasets/ (accessed on 15 March 2026).

## Abbreviations

The following abbreviations are used in this paper:

NIDS	Network Intrusion Detection System
IoT	Internet of Things
DDoS	Distributed Denial-of-Service
SiLU	Sigmoid Linear Unit
DWConv	Depthwise Convolution
MTC	Multi-Task Classification
LSTM	Long Short-Term Memory
CNN	Convolutional Neural Network
RNN	Recurrent Neural Network
GNN	Graph Neural Network
FFN	Feed-Forward Network
SSM	State Space Model
FPR	False Positive Rate
MCC	Matthews Correlation Coefficient
AUC	Area Under the Curve
SMOTE	Synthetic Minority Oversampling Technique
MLP	Multilayer Perceptron
FLOPs	Floating Point Operations
SRAM	Static Random Access Memory
DRAM	Dynamic Random Access Memory
OOM	Out-of-Memory
TPR	True Positive Rate
